# Memantine Prevents the WIN 55,212-2 Evoked Cross-Priming of Ethanol-Induced Conditioned Place Preference (CPP)

**DOI:** 10.3390/ijms22157940

**Published:** 2021-07-26

**Authors:** Marta Marszalek-Grabska, Irena Smaga, Paulina Surowka, Pawel Grochecki, Tymoteusz Slowik, Malgorzata Filip, Jolanta H. Kotlinska

**Affiliations:** 1 Department of Experimental and Clinical Pharmacology, Medical University of Lublin, 20-090 Lublin, Poland; marta.marszalek-grabska@umlub.pl; 2 Department of Drug Addiction Pharmacology, Maj Institute of Pharmacology, Polish Academy of Sciences, 31-343 Kraków, Poland; smaga@if-pan.krakow.pl (I.S.); mal.fil@if-pan.krakow.pl (M.F.); 3 Affective Cognitive Neuroscience Laboratory, Department of Pharmacology, Maj Institute of Pharmacology, Polish Academy of Sciences, 31-343 Kraków, Poland; surowka@if-pan.krakow.pl; 4 Department of Pharmacology and Pharmacodynamics, Medical University of Lublin, 20-093 Lublin, Poland; pa.grochecki@gmail.com; 5 Experimental Medicine Center, Medical University of Lublin, 20-090 Lublin, Poland; tymoteusz.slowik@tlen.pl

**Keywords:** cannabinoid, glutamate, CNR1, GRIN1, GRIN2A, ethanol relapse, rats

## Abstract

The activation of the endocannabinoid system controls the release of many neurotransmitters involved in the brain reward pathways, including glutamate. Both endocannabinoid and glutamate systems are crucial for alcohol relapse. In the present study, we hypothesize that N-methyl-D-aspartate (NMDA) glutamate receptors regulate the ability of a priming dose of WIN 55,212-2 to cross-reinstate ethanol-induced conditioned place preference (CPP). To test this hypothesis, ethanol-induced (1.0 g/kg, 10% *w*/*v*, i.p.) CPP (unbiased method) was established using male adult Wistar rats. After CPP extinction, one group of animals received WIN 55,212-2 (1.0 and 2.0 mg/kg, i.p.), the cannabinoid receptor 1 (CB1) agonist, or ethanol, and the other group received memantine (3.0 or 10 mg/kg, i.p.), the NMDA antagonist and WIN 55,212-2 on the reinstatement day. Our results showed that a priming injection of WIN 55,212-2 (2.0 mg/kg, i.p.) reinstated (cross-reinstated) ethanol-induced CPP with similar efficacy to ethanol. Memantine (3.0 or 10 mg/kg, i.p.) pretreatment blocked this WIN 55,212-2 effect. Furthermore, our experiments indicated that ethanol withdrawal (7 days withdrawal after 10 days ethanol administration) down-regulated the CNR1 (encoding CB1), GRIN1/2A (encoding GluN1 and GluN2A subunit of the NMDA receptor) genes expression in the prefrontal cortex and dorsal striatum, but up-regulated these in the hippocampus, confirming the involvement of these receptors in ethanol rewarding effects. Thus, our results show that the endocannabinoid system is involved in the motivational properties of ethanol, and glutamate may control cannabinoid induced relapse into ethanol seeking behavior.

## 1. Introduction

One of the main problems in treating addiction is the high rate of relapse to drug use [1,2]. Factors that can trigger relapse in humans include exposure to drug-associated environmental cues, stressors, re-experience of the drug and negative withdrawal symptoms [3,4]. In some cases, addicts start to abuse other substances after quitting their primary addiction. This phenomenon suggests a similar motivational mechanism of reward for both drugs and leads to relapse for a previously abused drug [5].

Marijuana (cannabis) and alcohol are the most popular drugs amongst recreational users, and a majority of primary cannabis abusers report alcohol use as the most common secondary substance of abuse [6,7,8,9]. Furthermore, clinical study indicates that alcohol, but not cigarettes, caffeine or non-cannabis illicit drugs substitution occurs during cannabis abstinence among users with a diagnosis of past alcohol abuse or dependence. Increased alcohol use was also associated with increased alcohol craving [6]. Moreover, several observational studies of individuals with alcohol use disorders (AUD) suggest that cannabis is used/abused during treatment for AUD. Such cannabis use reduces alcohol abstinence at the end of treatment [10]. 

Two cannabinoid receptors, CB1 and CB2 have so far been cloned and characterized, both belong to the class of G protein-coupled receptors. CB1 is located in the central nervous system (CNS) and peripheral tissues and CB2 appears mainly in the cells of the immune system [11] although it has now also been identified in brainstem, cortex and cerebellum neurons [12]. The psychoactive effects of cannabinoids are due to the activation of the CB1 receptors in the CNS. High levels of these receptors are found in the brain areas that are part of the mesocorticolimbic pathway, including the prefrontal cortex, hippocampus, olfactory bulb and nucleus accumbens (NAc). All of these are implicated in motivational and rewarding processes and are altered by cannabinoid compounds [13,14]. The main function of CB1 receptors is the regulation of neurotransmitter release such as, for example, glutamate, gamma-aminobutyric acid (GABA) or dopamine in the brain [15,16]. This function as a neuromodulator can be observed when CB1 receptors are activated by endogenous cannabinoids or exogenous synthetic molecules, such as the cannabinoid receptor agonist-WIN 55,212-2 [17].

In contrast, the psychoactive and physiological effects of alcohol (ethanol) are due to activation of several receptor systems. Among others, N-methyl-D-aspartate (NMDA) ionotropic glutamate receptors have been shown to be directly modulated by ethanol [18]. NMDA receptors are heteromultimeric ion channels that contain two obligatory GluN1 subunits and two GluN2 (A-D) subunits. The main function of the activation of this excitatory receptor is to permit the influx of Ca2+ into the cell. This facilitates the induction of long-term potentiation (LTP), a core element of learning and memory, leading, for example, to habit formation [19]. The transition from ethanol abuse to dependence, as well as the transition from ethanol dependence to ethanol abstinence may involve changes in the NMDA receptor subunit composition [20,21]. Like CB1 receptors, NMDA receptors are widely distributed throughout the brain, including the mesocorticolimbic pathway [22,23].

A possible functional interaction between the two systems (CB1 and NMDA complex) has been suggested, and evidence has been presented for the presence of CB1 receptors at glutamatergic terminals in areas related to addictive behavior [24,25]. The mechanism of CB1 receptor activation is complex. CB1 receptors are located on presynaptic glutamate neurons where they are activated by endocannabinoids released from postsynaptic neurons (diffuse in a retrograde fashion) [16]. Activation of the CB1 receptor inhibits presynaptic Ca2+ influx which in turn decreases the probability of neurotransmitters release, including glutamate [17]. Retrograde inhibition of neurotransmission has been reported for GABA and glutamate neurons throughout the whole brain [17] and individual brain structures [26,27,28].

Using complementary behavioral and biochemical methodologies in vivo and *in vitro*, the present study was designed to examine the relationship between ethanol and the endocannabinoids system, and the role of glutamate in this phenomenon. We sought to determine whether WIN 55,212-2, a synthetic CB1 agonist, given after the extinction phase in ethanol-conditioned rats can reinstate ethanol-induced CPP. Furthermore, considering that CB1 receptors regulate excessive glutamate neurotransmission, and that alcohol relapse is mediated by increases in glutamatergic signal transmission [29,30], we examined whether memantine, a NMDA receptor antagonist, could reverse the effect of WIN 55,212-2. In addition, a separate group of animals was used to perform a set of biochemical analyses to examine the effect of ethanol on the mRNA expression of the NMDA receptor subunits (GluNR1 and GluNR2A) and the CB1 receptors in the prefrontal cortex, hippocampus and dorsal striatum in ethanol withdrawal rats on the day before the reinstatement of the ethanol CPP paradigm by WIN 55,212-2. 

## 2. Results

### 2.1. The Effect of Ethanol and WIN 55,212-2 on the Reinstatement of Ethanol-CPP

In the presented experiment we assessed the effect of a priming dose of ethanol (1.0 g/kg, 10% *w*/*v*, i.p.) and WIN 55,212-2 (1.0 and 2.0 mg/kg, i.p.) on the reinstatement, performed 24 h following the last extinction trial of ethanol CPP in rats. 

**Figure 1:**
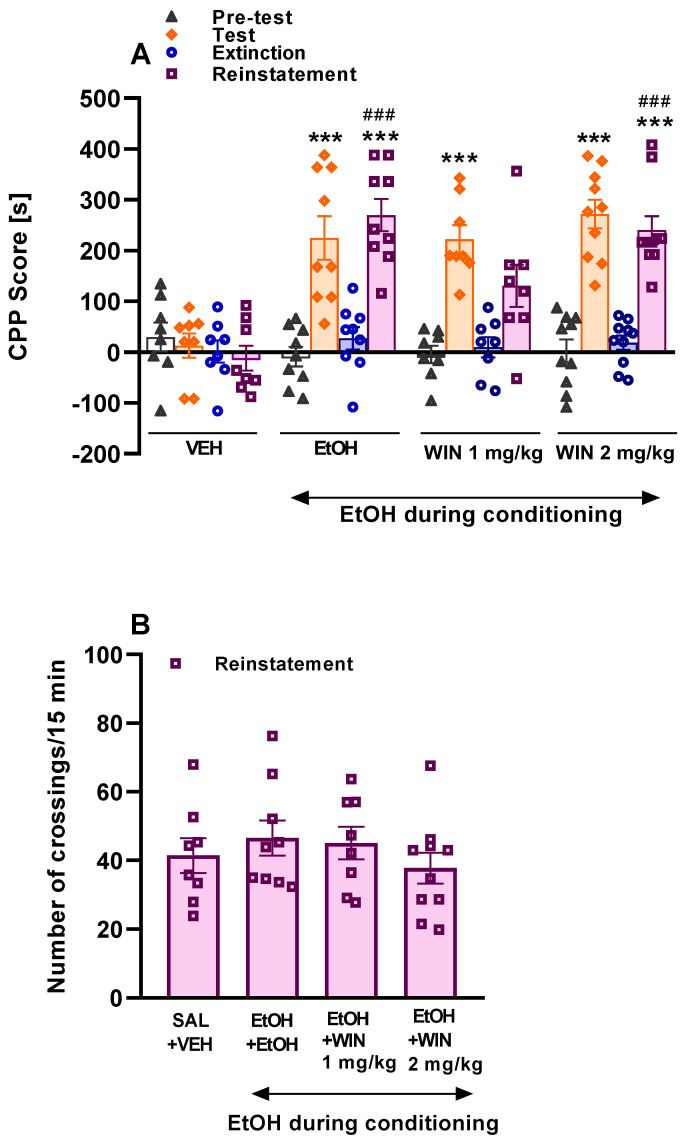
(**A**): Effect of ethanol and WIN 55,212-2 on the reinstatement of ethanol-induced CPP. Doses in each reinstatement: ethanol (EtOH) 1.0 g/kg; WIN 55,212-2 (WIN) 1.0 and 2.0 mg/kg. The bars represent the mean (±SEM) time spent in the drug-paired compartment before conditioning sessions (triangles), after conditioning sessions (diamonds), in the last extinction session (circles) and in the reinstatement test (squares). *** *p* < 0.001 significant difference in the time spent in pre-conditioning vs. post-conditioning test. ### *p* < 0.001 significant difference in the time spent in extinction vs. reinstatement test. EtOH: ethanol; WIN: WIN 55,212-2. (**B**): Locomotor activity measured as the number of crossings from one compartment to another during 15 min of the reinstatement (n = 8–10).

The two-way ANOVA revealed a significant effect of the test phase (F(3, 124) = 17.93, *p* < 0.0001); treatment (F(3, 124) = 49.97, *p* < 0.0001) and test phase x treatment interaction (F(9, 124) = 6.65, *p* < 0.0001) (Figure 1A). Our study indicated that the animals conditioned with ethanol (1.0 g/kg, 10% *w*/*v*) spent more time in a drug-paired compartment on the test day compared to the pre-test (*p* < 0.001). On the reinstatement day (one day after extinction trial), post hoc analysis (Tuckey’s test) showed that the priming dose of ethanol (1.0 g/kg, 10% *w*/*v*) given after the extinction phase in the ethanol-conditioned rats reinstated ethanol-induced CPP (*p* < 0.001). Furthermore, our results indicated that the priming injection of WIN 55,212-2 at a dose of 2.0 mg/kg, i.p., but not 1.0 mg/kg, i.p. reinstated (cross-reinstatement) the extinguished ethanol-induced CPP (*p* < 0.001) (Figure 1A).

We also evaluated the effect of a priming dose of ethanol (1.0 g/kg, 10% *w*/*v*, i.p.) and WIN 55,212-2 (1.0 and 2.0 mg/kg, i.p.) on the locomotor activity of rats measured as the number of crossings during 15 min of reinstatement. In the presented experiment, neither the priming dose of ethanol nor WIN 55,212-2 had an effect on the locomotor activity of rats (F(3,31) = 0.70, *p* > 0.05) (Figure 1B).

### 2.2. The Influence of Memantine on the Effect of WIN 55,212-2 on the Reinstatement of Ethanol CPP

In the presented experiment, we assessed the influence of memantine pretreatment (3.0 and 10 mg/kg, i.p.) on the priming dose of WIN 55,212-2 (2.0 mg/kg, i.p.) given on the reinstatement performed 24 h following the last extinction trial of ethanol CPP in rats.

**Figure 2:**
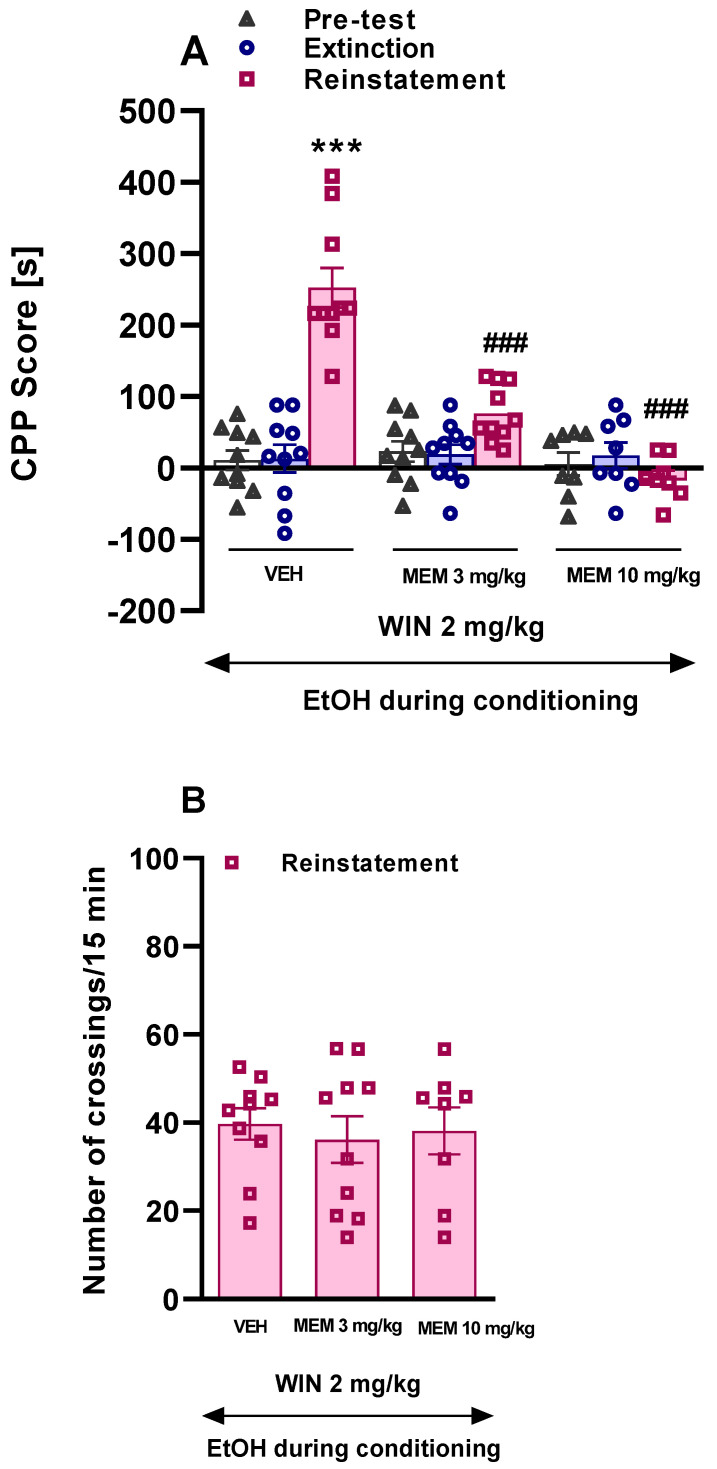
(**A**): Effect of memantine on the WIN 55,212-2 induced reinstatement of ethanol CPP. Doses in each reinstatement: Memantine (MEM) 3.0 and 10 mg/kg; WIN 55,212-2 (WIN) 2.0 mg/kg. The bars represent the mean (±SEM) time spent in the drug-paired compartment before conditioning sessions (triangles), in the last extinction session (circles) and in the reinstatement test (squares). (**B**): Locomotor activity measured as the number of crossings from one compartment to another during 15 min of the reinstatement. *** *p* < 0.001 vs. vehicle, ### *p* < 0.001 vs. WIN 55,212-2. EtOH: ethanol; WIN: WIN 55,212-2; MEM: memantine (n = 8–10).

The two-way ANOVA revealed a significant effect of the test phase (F(2, 75) = 26.90, *p* < 0.0001); treatment (F(2, 75) = 19.53, *p* < 0.0001) and test phase x treatment interaction (F(4, 75) = 20.51, *p* < 0.0001) (Figure 2A). Our study indicated that the priming dose of WIN 55,212-2 (2.0 mg/kg, i.p.) given after the extinction phase in the ethanol-conditioned rats, reinstated ethanol CPP (*p* < 0.001). Memantine at the dose of 3.0 and 10 mg/kg, i.p. pretreatment before priming dose of WIN 55,212-2 reversed the effect of WIN 55,212-2 (*p* < 0.001) (Figure 2A).

In the presented experiment, neither the priming dose of WIN 55,212-2 (2.0 mg/kg, i.p.) nor memantine (3.0 and 10 mg/kg, i.p.) had an effect on the locomotor activity of rats measured as the number of crossings during 15 min of reinstatement (F(2,25) = 0.86, *p* > 0.05) (Figure 2B).

### 2.3. CNR1 Gene Expression

**Figure 3:**
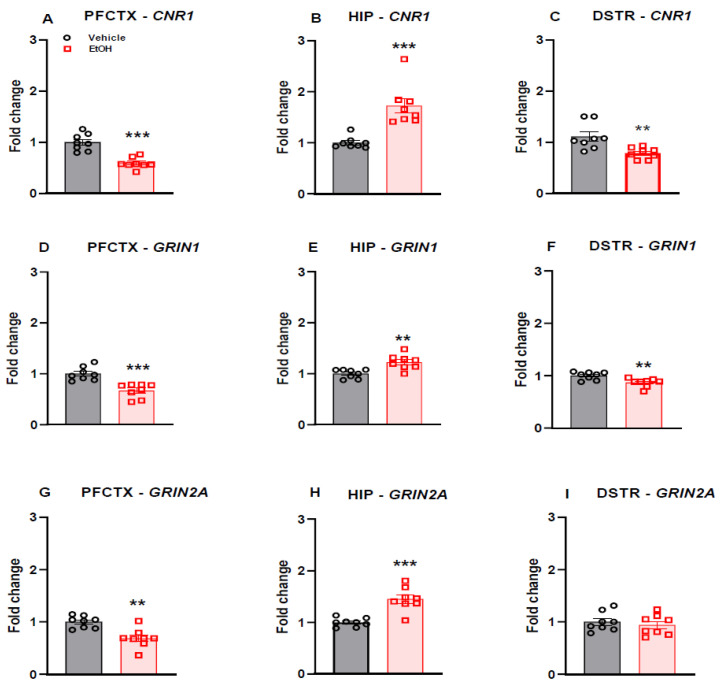
The effect of ethanol withdrawal on CNR1 gene expression in the prefrontal cortex (**A**), hippocampus (**B**) and dorsal striatum (**C**); GRIN1 gene expression in the prefrontal cortex (**D**), hippocampus (**E**) and dorsal striatum (**F**); GRIN2A gene expression in the prefrontal cortex (**G**), hippocampus (**H**) and dorsal striatum (**I**) of rats. Results are expressed as mean ± S.E.M. (n = 8). ** *p* < 0.01, *** *p* < 0.001 vs. vehicle. EtOH: ethanol; PFCTX: prefrontal cortex; HIP: hippocampus; DSTR: dorsal striatum.

A separated group of animals was examined to assess the effect of ethanol withdrawal on the day before the reinstatement of ethanol CPP paradigm by WIN 55,212-2 on the mRNA expression of the CB1 receptor in the prefrontal cortex, hippocampus and dorsal striatum. Our study revealed that cannabinoid receptor CNR1 mRNA level was either decreased in the prefrontal cortex (*p* < 0.001 by Student’s *t*-test; *p* < 0.0001) (Figure 3A) and in the dorsal striatum (*p* < 0.01 by Student’s *t*-test; *p* = 0.0056) (Figure 3C) or increased in the hippocampus (*p* < 0.001 by Student’s *t*-test; *p* = 0.0002) of rats following 7 days of ethanol withdrawal (Figure 3B). 

### 2.4. NMDA Receptor Subunit Gene Expression

The separated group of animals was examined to ascertain the effect of ethanol withdrawal on the day before the reinstatement of the ethanol CPP paradigm by WIN 55,212-2 on the mRNA expression of the NMDA receptor subunits (GluNR1 and GluNR2A) in the prefrontal cortex, hippocampus and dorsal striatum. Our study indicated that GRIN1 (*p* < 0.001 by Student’s *t*-test; *p* = 0.0003) (Figure 3D) and GRIN2A (*p* < 0.01 by Student’s *t*-test; *p* = 0.0011) (Figure 3G) mRNA levels were reduced in the prefrontal cortex of rats following 7 days of ethanol withdrawal, while the hippocampal levels of GRIN1 (*p* < 0.01 by Student’s *t*-test; *p* = 0.0019) (Figure 3E) and GRIN2A (*p* < 0.001 by Student’s *t*-test; *p* = 0.0001) (Figure 3H) were increased in these rats. In addition, GRIN1 mRNA level was reduced (*p* < 0.01 by Student’s *t*-test; *p* = 0.0043) in the dorsal striatum of these rats (Figure 3F). There was no significant effect of 7 days of ethanol withdrawal on GRIN2A gene expression level in the dorsal striatum (Figure 3I).

## 3. Discussion

The current study revealed that activation of CB1 receptors by a priming injection of WIN 55,212-2 dose dependently reinstates (cross-reinstates) the ethanol-induced CPP. The effectiveness of WIN 55,212-2 was similar to that of the priming dose of ethanol. Pretreatment with memantine, a NMDA receptor antagonist, prevented the effect elicited by WIN 55,212-2. Herein, WIN 55,212-2 and memantine did not have influence on locomotor activity during the reinstatement phase of CPP. In addition, our experiments showed that the expression of genes such as CNR1, GRIN1 and GRIN2A was decreased in the prefrontal cortex and dorsal striatum (except GRIN2A) but increased in the hippocampus at day 7 of ethanol withdrawal after 10 days of ethanol administration (the period comparable with extinction of ethanol CPP). Thus, our results demonstrate that the endocannabinoid system is involved in the rewarding/reinforcing properties of ethanol. Hence, a cannabinoid agonist can increase relapse to ethanol seeking behavior and an NMDA glutamate receptor antagonist may be useful in preventing this. 

It should be emphasized that these experiments were carried out on male rats. However, sex differences are present for all the phases of drug abuse in humans and animals [31]. For most age groups, men have higher rates of use or dependence on illicit drugs and alcohol than do women [9]. Still, women are just as likely as men to develop a substance use disorder [32]. In addition, women may be more susceptible to craving [33] and relapse [34,35], which are key phases of the addiction cycle. Therefore, we hypothesize that in females, acute WIN 55,212-2 administration could have more profound behavioral and biochemical effects on reinstatement of ethanol CPP than in males.

Endocannabinoid signaling plays an important role in regulation of alcohol consumption and reward, for example, [36,37,38,39,40,41,42]. There is evidence indicating that 2-arachidonoyl glycerol (2-AG), an endocannabinoid ligand, is released following administration of ethanol in the NAc, a brain structure crucial for drug reward [43,44]. The magnitude of 2-AD release is correlated with the amount of ethanol consumed in the self-administration procedure [44]. Thus, this data suggests that 2-AG release may, in part, encode the motivation to consume ethanol. 

Our study confirms the crucial role of the endocannabinoid system in ethanol effects. In our work, we indicated that at day 7 of withdrawal after 10 days ethanol administration (1.0 g/kg, 10% *w*/*v*, i.p., once a day), the CNR1 gene expression was up-regulated in the hippocampus and down-regulated in the prefrontal cortex and dorsal striatum. Several studies, however, suggest that CB1 returns to normal following 24 h of ethanol deprivation in chronic ethanol treated animals [45,46]. Rimondini et al., moreover, found that withdrawal from chronic ethanol exposure increased CB1 transcripts in the rat prefrontal cortex [47]. Yet other works suggest that withdrawal from chronic ethanol exposure induces increased CB1 protein in the hippocampus [48,49,50], striatum [49,50], and NAc [50] that persist for up to 40 days. Different effects of single versus repeated alcohol withdrawal on the expression of endocannabinoid system-related genes in the rat amygdala were also demonstrated [46]. These results suggest that that ethanol withdrawal is associated with dysregulation of CB1 signaling in the brain structures involved in reward behavior. 

However, the alteration in CB1 receptors may contribute to withdrawal-related dysregulation of glutamate neurotransmission, especially NMDA receptors, that seem to be crucial for ethanol seeking behavior [51]. Our study showed that at day 7 of abstinence, changes in GRIN1 (encoding GluN1) and GRIN2A (encoding GluN2A) gene expression were evident in such brain structures as the prefrontal cortex, hippocampus and dorsal striatum. In the prefrontal cortex, down-regulation of GRIN1 and GRIN2A gene expression was found, but in the dorsal striatum, the GRIN1 expression was down-regulated, but GRIN2A was not changed. Altogether, our results showed changes in mRNA levels of NMDA receptor subunits and CB1 receptors during ethanol deprivation. These changes are characteristic within the affected brain structures and could have an impact on ethanol motivation and reward. 

In the NAc glutamatergic projections from the cerebral cortex, amygdala and hippocampus, information is driven about external situations and internal emotional and physiological states, thus contributing to addiction by consolidating reward-driven behavior [52,53]. It seems that particularly the amygdala–accumbens pathway plays a crucial role in ethanol conditioning [51,54]. In the central amygdala (CeA), chronic ethanol exposure decreases CB1 receptor function at GABAergic synapses [55]. However, in the basolateral amygdala (BLA), ethanol administration potentiated glutamatergic neurotransmission [56]. The glutamatergic afferents to the NAc [57] control the firing of the NAc GABAergic neurons, which in turn inhibit the dopaminergic neurons in the ventral tegmental area. Via the reduction of excitatory neurotransmission in the NAc, cannabinoids could disinhibit dopamine cells of the ventral tegmental area, increase their firing rate, and trigger the release of dopamine in the NAc that is essential for the expression of the ethanol rewarding effects. Published data indicate that lifelong CB1 receptor deletion reduces ethanol-induced CPP and this effect is correlated with an overexpression of striatal dopamine D2 receptors [58].

The BLA is relatively abundant in CB1 receptors, and activation of this brain region (by endo- or exogenous cannabinoids) is a likely candidate to convey the indirect effects of cannabinoids on dopamine release in the NAc [59]. Thereby, CB1 receptor activation in the BLA could have been responsible for the ethanol-induced CPP and reinstatement, and for WIN 55,212-2-induced cross-reinstatement. Since these data suggest that, although NAc dopamine mechanisms may be involved in ethanol reward and relapse to ethanol seeking, the glutamate mediated NAc mechanism may constitute a final common pathway into which such dopamine mechanisms feed. 

We also demonstrated that WIN 55,212-2 given to control animals did not change rat behavior. The potentiation of ethanol relapse-like behavior induced by WIN 55,212-2 was not caused by nonspecific locomotor effects, since no effect was observed in the locomotor tests. Thus, our results demonstrated that the cross-reinstatement between WIN 55,212-2 and ethanol is characteristic only for rats with a history of ethanol use. Importantly, our data support those of Roberts et al. [60] who found that exposure to the cannabinoid WIN 55,212-2 during abstinence from ethanol can reproduce effects that can only be seen in alcohol-dependent animals, indicating that alterations in the endocannabinoid system could be causing an important regulation of alcohol addiction.

Ethanol craving is induced by a hyperglutamatergic state [20]. The endocannabinoid system controls the activity of NMDA receptors (by the CB1-NMDA complex that is associated by HINT1 protein) [61,62], preventing their overactivation and providing neuroprotection of neuronal cells against excitotoxicity. Cannabinoid-mediated control of NMDA receptor function has been observed in functional studies [63,64,65,66]. Several studies have indicated that cannabinoids oppose glutamatergic NMDA receptor function through various mechanisms, such as the pre-synaptic reduction of glutamate release into the cleft [67,68,69], or the inhibition of post-synaptic cannabinoid receptors, the signaling pathways of which may interfere with those of NMDA receptors [66,70]. However, for such activity, C-terminal sequences of the CB1 and of the GluNR1 subunit of the NMDA can establish direct physical interactions, and CB1 activation promotes the co-internalization of the GluN1 subunit for activating this complex. 

In our study, memantine pretreatment prevented the WIN 55,212-2 --induced reinstatement of ethanol CPP. So far, published data show that uncompetitive NMDA receptor antagonists can interact with GluN1 and GluN2 subunits in the NMDA receptor [71] and prevent cannabinoids from efficiently internalizing CB1 receptors, which rapidly desensitize at the plasma membrane, producing reduction in the cannabinoid effects [62]. Thus, we hypothesize that memantine, by binding to NR1 and NR2 subunits, can prevent CB1 receptors internalization, leading to reduction in WIN 55,212-2 reward. Thus, the CB1–NMDA complex does not contribute to cannabinoid mediated reward, although it is required for endocannabinoids to negatively regulate the function of the ionotropic glutamate receptor.

A limitation of our study is a lack of data concerning the expression of NMDA receptor subunits after a priming injection of WIN 55,212-2 and ethanol. Alén et al. [72], using a rat paradigm of relapse-like drinking showed that the NMDA/glycine receptor antagonist, L-701 prevented a long-lasting increase in alcohol consumption induced by WIN 55,212-2. Furthermore, L-701 prevented/reversed the WIN 55,212-2 -induced increase/decrease of mRNA CNR1 transcripts levels in brain areas associated with addiction and relapse, such as the hypothalamus, striatum, cingulated anterior and amygdala regions. This fact, together with the finding that NR1 mRNA expression was reduced after WIN 55,212-2 exposure in the amygdala, supports the functional interaction between endocannabinoid and glutamate systems [72]. 

The inhibitory effect of memantine on the WIN 55,212-2 -induced reinstatement of ethanol CPP can also be explained in terms of memory impairment. CB1 receptors abundantly expressed on the glutamatergic neurons regulate synaptic transmission by regulating the release of excitatory neurotransmitters like glutamate. CB1 receptor activation has been associated with induction of long-term synaptic depression (LTD) and LTP [73]. The use of memantine to block glutamate transmission should theoretically render WIN 55,212-2 to lose some of its functionality to induce LTD and LTP. Thus, the mechanism of memantine may not involve the receptor relationship suggested above, but instead be a consequence of blocking the effects of altered glutamate release by WIN 55,212-2.

In conclusion, our results indicate that endocannabinoids and NMDA glutamatergic neurotransmission are involved in ethanol addiction. Furthermore, an increase in glutamatergic release is important in alcohol craving and relapse. Memantine, the NMDA receptor antagonist, prevents relapse induced by cannabinoids. Probably, the interaction between GluN1/N2 subunits of the NMDA receptor and CB1 receptor is important in the relapse-like behavior induced by cannabinoids in ethanol dependent persons. Thus, pharmacological therapy based on glutamate antagonists could be useful intervention for alcoholics who use marijuana during withdrawal.

## 4. Materials and Methods

### 4.1. Animals

Male Wistar rats (OMD, Lublin, Poland) weighing 200–250 g, aged 8–9 weeks, were included in the present study. The animals were maintained under standard laboratory conditions (12 h light/dark cycle, room temperature 21 ± 1 °C) with free access to tap water and laboratory chow (Sniff Spezialdiäten GmbH, Soest, Germany). Each experimental group consisted of 8–10 animals. The rats were handled once a day for five days before the beginning of the behavioral experiments. Two cohorts were used in our study, one in behavioral experiments and the other in molecular analyses. All experiments were conducted according to the National Institute of Health Guidelines for the Care and Use of Laboratory Animals and to the European Community Council Directive for the Care and Use of Laboratory Animals of 22 September 2010 (2010/63/EU). The study was approved by the Local Ethics Committee, Lublin, Poland (No 49/2017). 

### 4.2. Drugs

Ethanol (95%, *w*/*v*, Polmos, Poznan, Poland) was diluted in saline (0.9% NaCl) to a concentration of 10% (*w*/*v*) and administered intraperitoneally (i.p.) at a dose of 1.0 g/kg. This ethanol dosage regimen was established in our previous study, wherein the dose of 1.0 g/kg conditioned place preference in rats [74]. Memantine hydrochloride (Tocris Bioscience, Bristol, UK) was dissolved in saline and administered i.p. at doses of 3.0 and 10 mg/kg. WIN 55,212-2 ((R)-(+)-[2,3-dihydro-5-methyl-3-(4-morpholinylmethyl) pyrrolo[1,2,3-de]-1,4-benzoxazin-6-yl]-1-naphthalenylmethanonone mesylate; Tocris Bioscience, Bristol, UK) was suspended in a 1% solution of Tween 80 (Sigma, St. Louis, MO, USA) in saline solution and administered i.p. at doses of 1.0 and 2.0 mg/kg. All agents were administered in a volume of 2 mL/kg. Control groups received saline or saline with Tween 80 injections in the same volume and by the same route.

### 4.3. CPP Apparatus

In the present study, eight chambers (60 cm × 35 cm × 30 cm) were used. Each of these consisted of two large compartments (25 cm × 35 cm) separated by removable guillotine doors from a small central gray area (10 cm × 10 cm). The walls of the two large compartments differed in color, with one having white walls, while the other one was black. To provide a tactile difference between the compartments, one of the compartments (white) had a smooth floor, while the other one (black) had a grid floor. The whole apparatus was cleaned thoroughly between each test procedure to neutralize the odor trails, and then wiped with dry paper towels. The boxes were kept in a soundproof room with a neutral masking noise and with a dim 40 lx illumination. Data were scored by video tracking software (Ugo Basile, Gemonio, Italy).

### 4.4. CPP Procedure

The CPP procedure was based on methods described previously [74,75], with minor modification. The CPP procedure (unbiased design) consisted of six different phases: habituation (1 day), pre-test (1 day), conditioning (10 days), test (1 day), extinction (7 days) and reinstatement (1 day) as described below. The amount of time spent in each compartment was measured during the pre-test phase. These results were used to separate animals into groups with approximately equal biases for each side. Moreover, an appropriate control group was used that underwent the same CPP procedure as the drug-treated rats.

Habituation and pre-conditioning test: The first and second phases of the experiment (days 1–2) were aimed at assessing primary place preference and consisted of measuring the time of residence in the two areas of the apparatus for 15 min. During these phases, the animals were placed separately in the central, small gray area with the guillotine doors removed to allow access to the entire apparatus. On the second day (i.e., the pre-conditioning test), the time spent by the rats in each of the two large compartments was measured in order to determine an initial preference that was equal to our unbiased experimental design. In the experimental setup that we used in our study, the animals did not show a significant preference for either of the compartments during this phase. No injections were given to rats during this time.

Conditioning: The third phase of the experiment consisted of two 30 min sessions per day, morning and afternoon, for 10 days. Here, the rats were randomly assigned to control and ethanol groups. In the morning session, the animals were injected with saline and confined in one compartment. The afternoon session was conducted with an interval of at least 4 h. In the afternoon session, control groups were injected with saline, while ethanol groups received ethanol (1.0 g/kg, 10% *w*/*v*, i.p.) and were conditioned via the opposite compartment. The guillotine doors separating the two areas, were closed. Injections were administered immediately before confinement in one of the two large compartments. A dose of 1.0 g/kg ethanol was chosen for conditioning because it produces reliable CPP in rats after 10 days of conditioning. The neutral zone was never used during conditioning. The expression of CPP was conducted at approximately 24 h after the last conditioning session. Rats were given free access to the experimental compartments for 15 min, during which the amount of time spent in each of the two large compartments was recorded as described above for the pre-conditioning test.

Extinction and reinstatement: After post-conditioning, animals entered the extinction phase (7 days, one trial per day without any injection). During this phase, all animals were allowed free access to all compartments for 15 min on consecutive days until the time spent in the saline- and drug-paired compartments became like those of the pre-conditioning phase. Time spent in each compartment was recorded.

#### 4.4.1. The Effect of Ethanol and WIN 55,212-2 on the Reinstatement of Ethanol CPP

Once the CPP was extinguished, the reinstatement test was performed 24 h following the last extinction trial. On the reinstatement, the animals were given a priming dose of saline (control) or ethanol (1.0 g/kg, 10% *w*/*v*, i.p.) or WIN 55,212-2 (1.0 or 2.0 mg/kg, i.p.) and allowed to explore all compartments of the apparatus for 15 min. The time spent in each compartment was recorded.

#### 4.4.2. The Influence of Memantine Pretreatment on the Effect of WIN 55,212-2 on the Reinstatement of Ethanol CPP

In a separate group of rats conditioned with ethanol (1.0 g/kg, 10% *w*/*v*, i.p.), the influence of memantine on the effect of WIN 55,212-2 on the reinstatement of ethanol CPP was investigated. On reinstatement day, the selected groups of rats were treated with memantine (3.0 or 10 mg/kg, i.p.) 15 min before WIN 55,212-2 (2.0 mg/kg, i.p.) and allowed to explore all compartments of the apparatus for 15 min. The time spent in each compartment was recorded.

#### 4.4.3. The Effect of WIN 55,212-2 and Memantine on Locomotor Activity

The locomotor activity of individual rats was measured as the number of crossings from one compartment to another through the central grey area within 15 min of reinstatement.

### 4.5. Molecular Analyses

#### 4.5.1. RNA Isolation

RNA was isolated and purified using the RNA Mini Kit (A&A Biotechnology, Gdansk, Poland) following the manufacturer’s protocol. The quantity of the RNA was checked with a NanoDrop ND-1000 Spectrophotometer (Thermo Fisher Scientific, Waltham, MA, USA). RNA quality was determined using agarose gel electrophoresis, and RNA integrity was analyzed with chip-based capillary electrophoresis with an RNA 6000 Nano Chip Kit and an Agilent Bioanalyzer (Agilent Technologies, Santa Clara, CA, USA).

#### 4.5.2. RT-qPCR Analyses

Reverse transcription into cDNA was performed using a High-Capacity cDNA Reverse Transcription Kit (Thermo Fisher Scientific, Waltham, MA, USA, Life Technologies, Waltham, MA, USA). RT-qPCR was performed in duplicate on a 96-well plate using Quant Studio 3 (Thermo Fisher Scientific, Waltham, MA, USA) and TaqMan Gene Expression Assays (Applied Biosystems, San Francisco, CA, USA) for CNR1 (Rn00562880_m1), GRIN1 (Rn01436034_m1), and GRIN2A (Rn00561341_m1). The PCR cycling conditions were as follows: an initial step of 95 °C for 10 min, followed by 40 cycles of 95 °C for 15 s and then 60 °C for 60 s. Hypoxanthine phosphoribosyltransferase 1 (HPRT1) was used as a housekeeping control (Rn01527840_m1). The values are expressed as the fold change relative to the control (n = 8 rats/group).

### 4.6. Statistical Analysis

The data collected in the CPP test were expressed as means ± SEM of preference scores (i.e., the differences between post-conditioning and pre-conditioning time spent in the drug paired compartment). To evaluate the acquisition, extinction and reinstatement of CPP, a two-way analysis of variance (ANOVA) was used to determine the effects of WIN 55,212-2 or memantine dose (treatment), pretreatment (ethanol vs. 0.9% NaCl), or interaction between these factors, followed by the Tukey’s test to compare each group to the control group. One-way analysis of variance (ANOVA) with the Tukey’s post-test was applied to analyze the effect of WIN 55,212-2 and memantine on rat locomotor activity. In molecular analyses, specific paired comparison was performed with Student’s *t*-test. Statistical significance was set at *p* < 0.05. All data were performed using GraphPad Prism 8.0 Software, San Diego, CA, USA.

## Data Availability

The data presented in this study are available on request from the corresponding author.
